# Transcription factor LSF (TFCP2) inhibits melanoma growth

**DOI:** 10.18632/oncotarget.6230

**Published:** 2015-10-25

**Authors:** Yuji Goto, Ichiro Yajima, Mayuko Kumasaka, Nobutaka Ohgami, Asami Tanaka, Toyonori Tsuzuki, Yuji Inoue, Satoshi Fukushima, Hironobu Ihn, Mikiko Kyoya, Hiroyuki Ohashi, Tamihiro Kawakami, Dorothy C. Bennett, Masashi Kato

**Affiliations:** ^1^ Department of Biomedical Sciences, College of Life and Health Sciences, Chubu University, Matsumoto-cho, Kasugai-shi, Aichi, Japan; ^2^ Department of Biology, Faculty of Science, Toho University, Miyama, Funabashi, Japan; ^3^ Department of Occupational and Environmental Health, Nagoya University Graduate School of Medicine, Tsurumai-cho, Showa-ku, Nagoya, Aichi, Japan; ^4^ Department of Pathology, Nagoya Daini Red Cross Hospital, Nagoya, Aichi, Japan; ^5^ Department of Dermatology and Plastic Surgery, Faculty of Life Sciences, Kumamoto University, Kumamoto, Japan; ^6^ Department of Dermatology, St. Marianna University School of Medicine, Sugao, Miyamae-ku, Kawasaki, Kanagawa, Japan; ^7^ Molecular Cell Sciences Research Centre, St George's, University of London, London, UK

**Keywords:** melanoma, transcription factor LSF, TFCP2, CDKN1A, cell cycle

## Abstract

Late SV40 factor 3 (LSF), a transcription factor, contributes to human hepatocellular carcinoma (HCC). However, decreased expression level of LSF in skin melanoma compared to that in benign melanocytic tumors and nevi in mice and humans was found in this study. Anchorage-dependent and -independent growth of melanoma cells was suppressed by LSF overexpression through an increased percentage of G1 phase cells and an increased p21^CIP1^ expression level *in vitro* and *in vivo*. Anchorage-dependent growth in LSF-overexpressed melanoma cells was promoted by depletion of LSF in the LSF-overexpressed cells. Integrated results of our EMSA and chromatin immunoprecipitation assays showed binding of LSF within a 150-bp upstream region of the transcription start site of *p21*CIP1 in melanoma cells. Taken together, our results suggest potential roles of LSF as a growth regulator through control of the transcription of p21^CIP1^ in melanocytes and melanoma cells as well as a biomarker for nevus.

## INTRODUCTION

Approximately 50% of human melanomas have *BRAF* mutations [1]. In the most common *BRAF* mutation (90% of cases), valine 600 is substituted by glutamic acid (V600E) [2]. A previous study suggested that the expression of oncogenic BRAF^V600E^ in various benign lesions including human nevus cells contributes to their stable growth arrest of oncogene-induced senescence (OIS) [3].

We previously established Metallothionein-I/*RFP-RET* transgenic mice (RET-mice) that spontaneously develop systemic skin melanosis, benign melanocytic tumors and melanoma metastasizing to distant organs [4, 5]. Both RET-mice and a Mel-ret murine melanoma cell line from the tumor of a RET-mouse [6] might be strong tools for analyzing the molecular mechanism of melanoma growth.

Recent studies have shown that Late SV40 factor 3 (LSF), a transcription factor, functions as an oncogene in hepatocellular carcinomas (HCC) [7-9]. Previous studies suggest that increased expression level of LSF promotes malignant progression. In this study, we not only found opposite roles of LSF in melanoma compared to those previously reported in HCC but also revealed a novel molecular mechanism of LSF in melanocytic cells in mice and humans.

## RESULTS

### LSF expression levels in tumors of RET-mice

**Figure 1:**
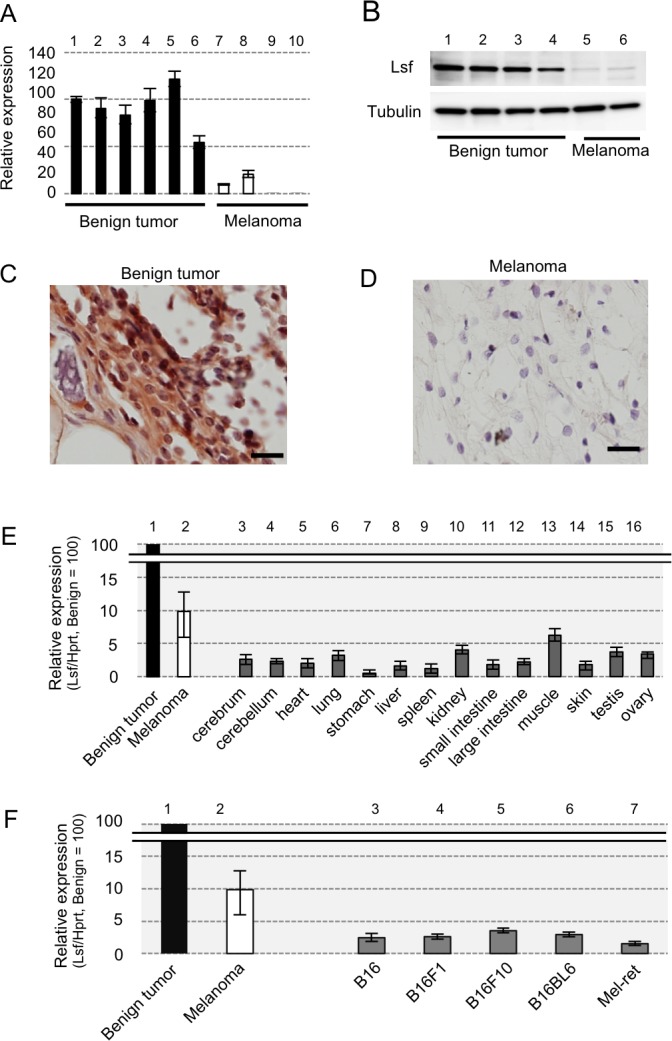
Lsf expression levels in mice **A.**-**F.** Results of RT-qPCR **A.**, **E.**, **F.**, immunoblot **B.** and immunohistochemical **C.**, **D.** analyses for benign melanocytic tumors (lanes 1-6 in **A.**, lanes 1-4 in **B.**, and **C.**, lane 1 in **E.** and **F.**) and melanomas (lanes 7-10 in **A.**, lanes 5-6 in **B.**, **D.**, lane 2 in **E.** and **F.**) from RET-mice, for indicated organs from wild-type mice (lanes 3-16 in **E.**), and for melanoma cell lines (lanes 3-7 in **F.**) of B16 and Mel-ret [6]. Hprt (**A.**, **E.**, **F.**) and α-Tubulin **B.** were used as internal controls in RT-qPCR and immunoblot analyses, respectively. Results (mean ± SD) are representative of three independent experiments.

Corresponding to our results of preliminary DNA microarray analysis using a benign melanocytic tumor and a primary melanoma in RET-mice, our RT-qPCR analysis showed that levels of *Lsf* transcript expression in benign tumors from RET-mice (lanes 1-6 in Figure 1A) were about 3-12-fold higher than those in melanomas from RET-mice (lanes 7-10 in Figure 1A). Lsf protein expression was detectable in benign tumors from RET-mice (lanes 1-4 in Figure 1B and 1C), but the expression in melanomas from RET-mice was undetectably low (lanes 5 and 6 in Figure 1B and Figure 1D). In addition, levels of *Lsf* transcript expression in normal murine tissues (lanes 3-16 in Figure 1E) and other murine melanoma cells including B16 cells and Mel-ret cells (lanes 3-7 in Figure 1F) were lower than those in benign melanocytic tumors and melanoma from RET-mice (lane 2 in Figure 1E and 1F), whereas *Lsf* has been reported to be ubiquitously expressed in normal mouse tissues [10]. These results suggest that Lsf expression level in melanoma is lower than that in benign melanocytic tumors in mice.

### Levels of LSF expression in human nevi and melanomas

**Figure 2:**
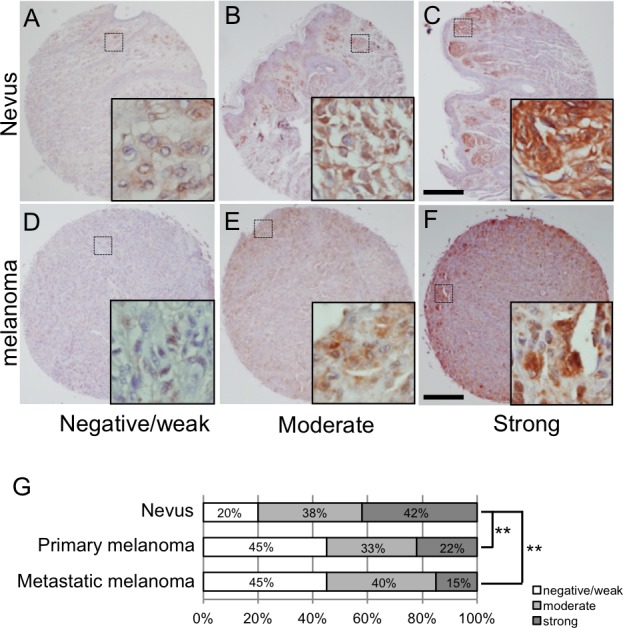
LSF expression levels in melanocytic tumors in humans **A.**-**F.** Representative signal scores for both nevus cell nevus **A.**-**C.** and melanoma **D.**-**F.** samples are shown as negative/weak (**A.** and **D.**), moderate (**B.** and **E.**) and strong (**C.** and **F.**). Dotted squares show the areas magnified in the inset. Scale bar, 200 μm. **G.** Percentages classified as negative/weak (white), moderate (light gray) and strong (dark gray) LSF protein expression levels in nevi and primary and metastatic melanomas are shown. Significantly different (**, *p* < 0.01) from nevi by Fisher's exact test.

Levels of LSF protein expression were examined in 24 nevus cell nevi, 55 primary melanomas and 20 metastatic melanomas in lymph nodes of humans. After confirming that LSF protein was expressed in nevus and melanoma cells but not in stroma cells (Figure S1), all of the samples were classified into three groups (weak/negative, moderate and strong) by the signal intensity of LSF protein (Figure 2A-2F), according to the method previously reported [11, 12]. As shown in Figure 2G, 42% of nevus cell nevi, 22% of primary melanomas and 15% of metastatic melanomas were classified as strong intensity. Statistical analysis by Fisher's exact test showed a significantly (*p* < 0.01) decreased expression level of LSF protein in primary and metastatic melanomas compared to that in nevus cell nevi. These results again suggest a lower level of LSF expression in melanoma compared to that in benign melanocytic tumors in humans.

### LSF overexpression-mediated G1/S arrest in melanoma cells *in vitro*

**Figure 3:**
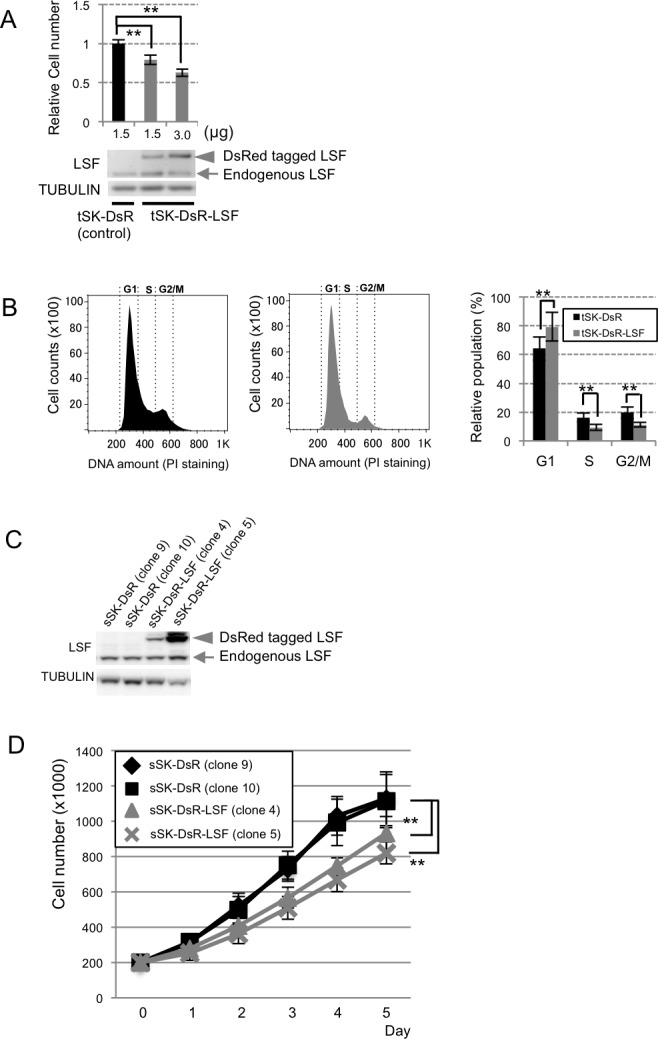
Effect of *LSF* overexpression on anchorage-independent growth of SK-Mel28 melanoma cells *in vitro* **A.** Levels of anchorage-dependent growth (mean ± SD) in control tSK-DsR and tSK-DsR-LSF cells, which had been transiently transfected with the indicated amounts of an empty vector and DsRed-LSF expression vector, respectively, were quantified by crystal violet staining after confirming LSF protein expression levels by immunoblot analysis. **B.** Results of cell cycle analysis by flow cytometry in control tSK-DsR cells (black bars) and tSK-DsR-LSF cells (gray bars) are shown. Histograms and a graph showing percentages (mean ± SD) of G1, S and G2/M phases are shown. **C.**, **D.** Immunoblot detection of LSF expression **C.** and anchorage-dependent growth (mean ± SD) **D.** in control tSK-DsR cells (black bars) and tSK-DsR-LSF cells (gray bars), which had been stably transfected with an empty vector and DsRed-LSF expression vector, respectively. Arrow, endogenous LSF; arrowhead, DsRed-fused LSF. Significantly different (**, *p* < 0.01) from the control by Student's t-test.

Anchorage-dependent growth was examined in human SK-Mel28 melanoma (tSK-DsR-LSF) cells transiently overexpressing DsRed-LSF fusion protein to elucidate the molecular function. Our cell proliferation assay using crystal violet showed that anchorage-dependent growth in tSK-DsR-LSF cells was decreased by about 19-40% compared to that in control human SK-Mel28 melanoma (tSK-DsR) cells transiently overexpressing DsRed protein (Figure 3A). Flow cytometric analysis showed that 62% in G1 phase of control tSK-DsR cells was increased to 80.5% in G1 phase of tSK-DsR-LSF cells (Figure 3B). Correspondingly, anchorage-dependent growth of human SK-Mel28 melanoma (sSK-DsR-LSF) cells stably overexpressing DsRed-LSF fusion protein was significantly suppressed compared to that of control human SK-Mel28 melanoma (sSK-DsR) cells stably overexpressing DsRed protein (Figure 3C and 3D). Then 3 kinds of LSF-depleted sSK-DsR-LSF cells transfected with 3 kinds of siRNAs for *LSF* and control NG-sSK-DsR-LSF cells transfected with negative control siRNA were developed. After confirming decreased levels of LSF transcript and protein expression in LSF-depleted sSK-DsR-LSF (1), (2) and (3) cells compared to those in NG-sSK-DsR-LSF cells (Figure S2A, S2B), anchorage-dependent growth was examined. As expected, the level of anchorage-dependent growth of LSF-depleted sSK-DsR-LSF (2) cells was significantly higher than those of sSK-DsR-LSF cells and NG-sSK-DsR-LSF cells and was near to that of sSK-DsR cells (Figure S2C).

Correspondingly, the percentage of G1 phase cells in murine B16F10 melanoma (tB16-FLAG-LSF) cells transiently overexpressing FLAG-LSF fusion protein was higher than that in control murine B16F10 melanoma (tB16-FLAG) cells transiently overexpressing FLAG protein (Figure S3A, S3B). Anchorage-dependent growth of murine B16F10 melanoma (sB16-FLAG-LSF) cells stably overexpressing FLAG-LSF fusion protein was again decreased compared to that of control murine B16F10 melanoma (sB16-FLAG) cells stably overexpressing FLAG protein (Figure S3C, S3D).

### Effect of LSF overexpression on anchorage-independent growth of melanoma cells

**Figure 4:**
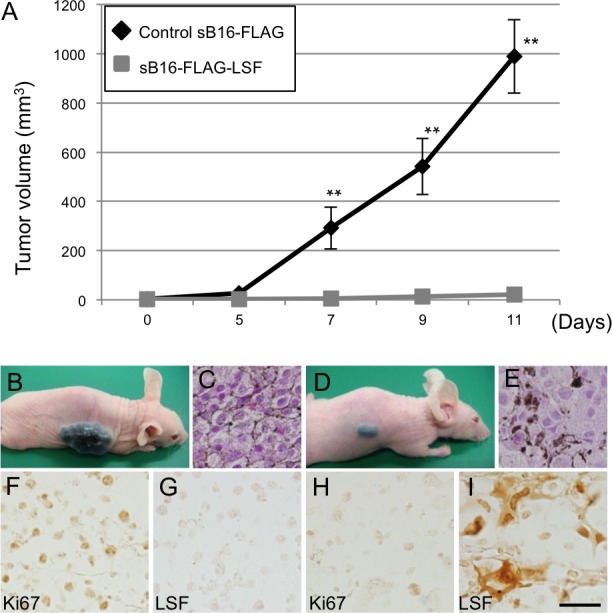
Effect of *LSF* overexpression on anchorage-independent growth of B16F10 melanoma cells *in vivo* **A.**-**I.** Tumor volumes **A.** and macroscopic (**B.**, **D.**) and microscopic (**C.**, **E.**, **F.**-**I.**) appearances in nude mice at 11 days after subcutaneous inoculation of control B16F10 melanoma cells (sB16-FLAG; n=5) (**B.**, **C.**, **F.**, **G.**) and B16F10 cells stably overexpressing LSF (sB16-FLAG-LSF; n=6) (**D.**, **E.**, **H.**, **I.**). Results of HE staining (**C.**, **E.**) and immunohistochemical detection of Ki67 (**F.**, **G.**) and LSF (**H.**, **I.**) for the inoculated melanoma are presented. Significantly different (**, *p* < 0.01) from the control by Student's t-test. Scale bar, 100 μm.

Since inoculated sSK-DsR-LSF cells and control sSK-DsR cells did not form subcutaneous tumors in nude mice within two months, nude mice were inoculated with LSF-overexpressed sB16-FLAG-LSF cells and control sB16-FLAG cells. Tumor volume of inoculated LSF-overexpressed cells (*n* = 6) was less than 1% of that of control cells (*n* = 5) (Figure 4A-4E). Immunohistochemical detection of Ki67 showed that the number of proliferating cells in LSF-overexpressed cells was decreased compared to that in control cells (Figure 4F, 4H). The level of angiogenesis in tumors derived from LSF-overexpressed cells was lower than that in tumors derived from control cells (Figure S4). These results showing increased LSF-mediated decrease in angiogenesis in melanoma may simply reflect the difference in tumor size between sB16-FLAG-LSF cells and sB16-FLAG cells. Further study is needed to clarify the direct effect of LSF on angiogenesis in melanoma. Our colony formation assay *in vitro* showed that anchorage-independent growth of LSF-overexpressed sB16-FLAG-LSF cells was suppressed compared to that of control sB16-FLAG cells (Figure S5A). Expression and phosphorylation levels of FAK and AKT protein were comparable between LSF-overexpressed sB16-FLAG-LSF cells and control sB16-FLAG cells (Figure S5B). These results suggest that the contribution of FAK and AKT activities to LSF-mediated anchorage-independent growth in melanoma is limited.

### Increased cell-cycle inhibitors by LSF overexpression

**Figure 5:**
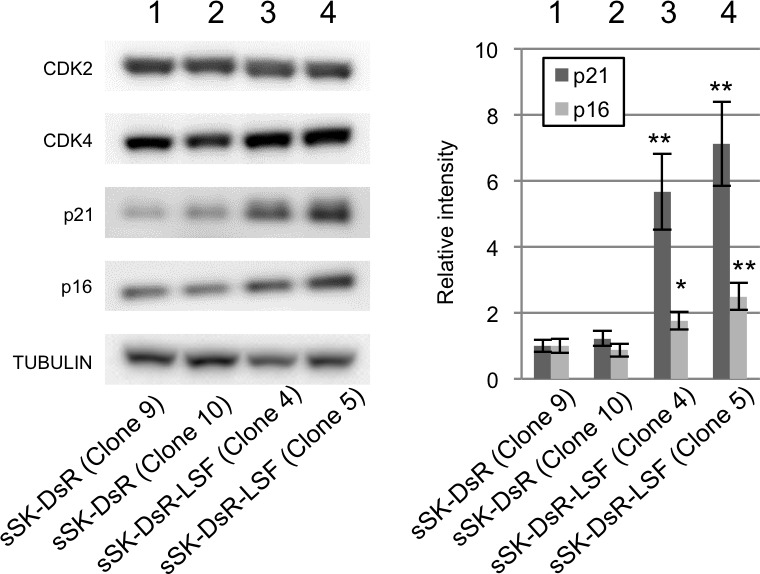
Effect of *LSF* overexpression on cell cycle regulators in melanoma cells Representative results of immunoblot analysis for expression levels of cell cycle regulators, CDK2, CDK4, p21^CIP1^and p16^INK4a^, in SK-Mel28 cells stably overexpressing DsRed protein (sSK-DsR) (lanes 1 and 2) and DsRed-LSF fusion protein (sSK-DsR-LSF) (lanes 3 and 4) are presented. α-TUBULIN served as an internal control. Graph of relative intensities (means ± SD) of p21^CIP1^and p16^INK4a^ protein expression levels obtained from three independent experiments are presented. Significantly different (**, *p* < 0.01; *, *p* < 0.05) from the control [sSK-DsR (Clone 9)] in each analysis of p21^CIP1^and p16^INK4a^ by Student's t-test.

To analyze the molecular mechanism of LSF-mediated growth in melanoma, expression levels of four major players in cell cycle regulation were examined in LSF-overexpressed sSK-DsR-LSF cells and control sSK-DsR cells (Figure 5). Although protein expression levels of CDK2 and CDK4 were comparable between LSF-overexpressed and control cells, protein expression levels of p21^CIP1^ and p16^INK4a^ in LSF-overexpressed cells were 5.7-7.1-fold and 1.9-2.2-fold higher than those in control cells, respectively (Figure 5). The increased level of p21^CIP1^ protein in LSF-overexpressed sSK-DsR-LSF cells was decreased in LSF-depleted sSK-DsR-LSF cells but not in NG-sSK-DsR-LSF cells (Figure S2D).

### LSF-mediated regulation of p21^CIP1^ transcription in melanoma cells

**Figure 6:**
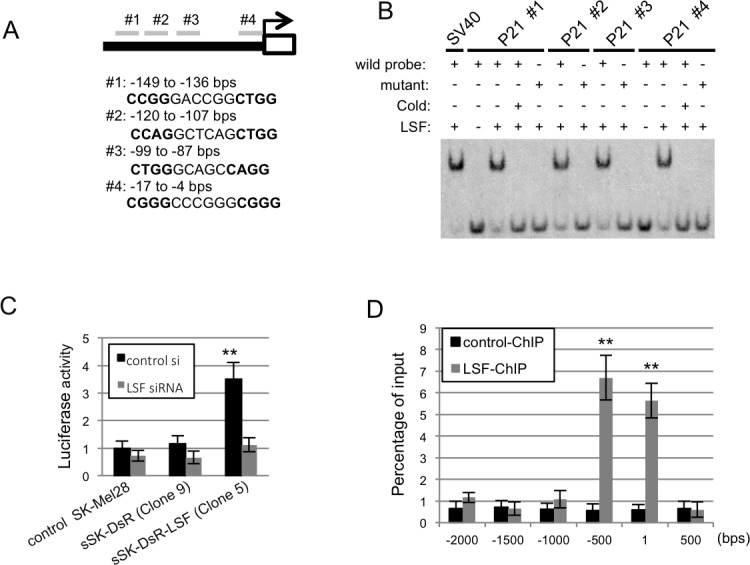
Association of LSF with *p21^CIP1^* promoter sequences *in vitro* and *in vivo* **A.** Putative CNRG-N_6_-CNRG motifs in the region of −149 to −136 bps (#1), −120 to −107 bps (#2), −99 to −87 bps (#3) and −17 to −4 (#4) bps from the transcription start site of *p21^CIP1^* are shown. **B.** Results of the EMSA assay using putative LSF-binding (wild) and mutant DNA probes in the presence or absence of *in vitro* translated LSF and competitor oligonucleotides (cold) are shown. **C.** Luciferase activity (mean ± SD) by binding of *LSF* in the promoter region of *p21^CIP1^* in control parental SK-Mel28 cells, sSK-DsR cells and sSK-DsR-LSF cells in the presence (gray) or absence (black) of transient depletion of *LSF*. **D.** The LSF binding of each region in the immunoprecipitates was evaluated by qPCR, and the results are expressed as percentage (mean ± SD) to the input. Significantly different (**, *p* < 0.01) from the control by Student's t-test.

The transcription factor LSF interacts with the CNRG-N_6_-CNRG motif in target DNAs [13-16]. Scanning of the *p21^CIP1^* sequence revealed four similar motifs from the transcription start site to the 150-bp upstream region (Figure 6A), whereas the motif was not present in the *p16^INK4A^* promoter. These results suggest that LSF regulates the transcription of *p21^CIP1^*by binding to its promoter region. Our results of EMSA using purified recombinant FLAG-tagged human LSF showed that LSF binds to all of the four candidate regions (Figure 6B). Binding of LSF using probes #1 and #4 was sequence-specific because the band-shift was abolished by competition with cold oligonucleotides and by substitution of mutated probes.

To determine whether LSF can induce *p21^CIP1^* transcription, we cloned a 1.2-kb region in the *p21^CIP1^* promoter containing the putative LSF binding sites into a luciferase reporter vector. This construct was transfected to parental SK-Mel28 cells and LSF-overexpressed sSK-DsR-LSF cells. As shown in Figure 6C, luciferase activity in the LSF-overexpressed cells was increased compared to that in control parental SK-Mel28 cells and in SK-Mel28 cells transfected with an empty vector. Co-transfection of the *LSF* siRNA with the reporter construct abolished the elevation of luciferase activity, suggesting functional relevance of LSF-mediated transactivation of *p21^CIP1^*.

Chromatin immunoprecipitation (ChIP) was performed to analyze the association of LSF with the promoter region of *p21^CIP1^ in vivo* in sSK-DsR-LSF cells. Cross-linked chromatin was precipitated with an antibody against DsRed followed by qPCR using site-specific primers against the putative LSF binding sites within the *p21^CIP1^* promoter region. Levels of LSF binding from the transcription start site to the 500-bp upstream region were 5.9-12.2-fold higher than those in other regions (Figure 6D). These results suggest that LSF regulates *p21^CIP1^* transcription by directly binding to specific regions of the *p21^CIP1^* promoter.

### LSF-mediated regulation of p21^CIP1^ transcription in melanocytes

**Figure 7:**
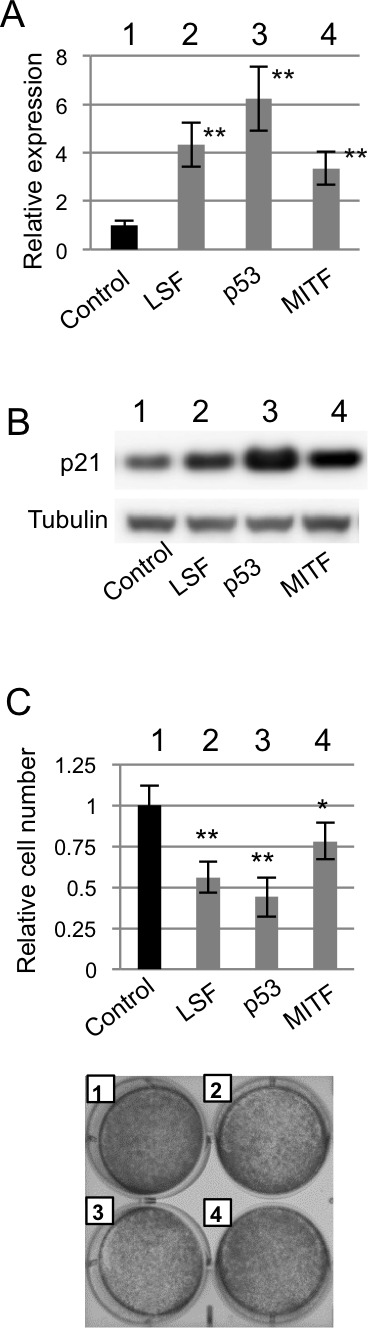
Effect of *LSF* overexpression on anchorage-dependent growth of melanocytes *in vitro* (**A.**-**C.**) Levels of p21^CIP1^ transcript **A.** and protein **B.** expression and anchorage-dependent growth on day 3 **C.** in control murine melan-a melanocytes (lane 1 in **A.**-**C.**) and melan-a melanocytes transiently overexpressing LSF (lane 2 in **A.**-**C.**), p53 (lane 3 in **A.**-**C.**) and MITF (lane 4 in **A.**-C.). Results (means ± SD) from three independent experiments are shown in the graphs (**A.**, **C.**). Results of crystal violet staining are shown in the photographs **C..** Significantly different (**, *p* < 0.01; *, *p* < 0.05) from the control by Student's t-test.

Lastly, the effect of LSF expression on anchorage-dependent growth was examined. Transfection efficiency was undetectably low in NHEM. Therefore, murine immortalized normal melanocytes (melan-a) were used (Figure 7). A previous study showed that p21 transcription was regulated by p53 and MITF [17]. After confirming that transcript and protein expression levels of p21 were increased in melan-a cells transiently overexpressing LSF, p53 and MITF compared to those in control melan-a cells (Figure 7A and 7B), anchorage-dependent growth was examined (Figure 7C). Anchorage-dependent growth was decreased in melan-a cells transiently overexpressing LSF, p53 and MITF compared to that in control melan-a cells (Figure 7C). These results suggest that LSF is associated with anchorage-dependent growth of normal melanocytes as well as melanoma cells.

## DISCUSSION

In this study, we first showed that the expression level of LSF in melanoma was decreased compared to that in nevi. We then showed that increased LSF expression levels suppressed anchorage-dependent growth and -independent growth of melanoma cells *via* regulation of the cell cycle with increased p21^CIP1^ and p16^INK4a^ expression levels *in vitro* and *in vivo*. We finally provided evidence of binding of *LSF* in the promoter region of *p21^CIP1^* in melanoma.

At present, only a handful of markers have been identified for benign melanocytic tumors including nevi [18]. Spitz nevus and dysplastic nevus were also classified as strong intensity in accordance with the classification method in Figure 2 (Figure S6). *LSF* transcript expression levels in nevus cell nevi were 1.6-5.1-fold higher than those in melanomas and various tissues in humans (Figure S7). Thus, LSF is a potential diagnostic marker for benign melanocytic tumors in humans.

Previous studies showed independent pathways of p16^INK4a^ and p21^CIP1^ in BRAF^V600E^-mediated OIS in melanoma [3]. DNA-binding activity of LSF has been reported to be controlled *via* phosphorylation of LSF at serine 291 by ERK, which is potentially sited downstream of BRAF^V600E^ [19, 20]. Taken together, our results showing an LSF-mediated increase in p21^CIP1^ expression level in melanocytic cells suggest that LSF contributes to an OIS pathway through the pathway of BRAF^V600E^/ERK/LSF/p21^CIP1^ (Figure S8).

In conclusion, we newly propose a potential role of LSF in melanocytic cells as a growth regulator as well as a biomarker.

## MATERIALS AND METHODS

### Mice, cell lines and culture conditions

Previously established RET-mice of line 304/B6 [4, 5] were used. Normal human epithelial melanocytes (NHEM) were purchased from KURABO Co. and were maintained in melanocyte growth medium containing hydrocortisone and growth supplements. G361 cells were provided by Cell Resource Center for Biomedical Research, Tohoku University. Human SK-Mel28 melanoma cells and mouse B16 melanoma cell lines were purchased from the Riken Bioresource Center Cell Bank. The immortal melanocyte cell line melan-a was provided by the Wellcome Trust Cell Bank at St George's, University of London. This cell line was cultured in RPMI-1640 medium supplemented with 10% fetal bovine serum (FBS) and 200 nM 12-o-tetradecanoyl phorbol-13-acetate (Sigma, USA). Other cell lines were maintained in DMEM supplemented with 10% FBS in a 5% CO_2_ atmosphere.

### Reverse transcription and quantitative PCR

RNA extraction and cDNA synthesis were performed as described previously [21]. Quantitative PCR was performed with FastStart Universal SYBR Green Master with Rox (Roche) on a 7500 FAST (Applied Biosystems). Each PCR assay was run in triplicate for checking PCR variations. Expression levels of the target genes were normalized to that of hypoxanthine guanine phosphoribosyl transferase (*Hprt*). The primers used were: human *LSF*, tggcttcaacagttcccata and tctggctggtggtttggt; mouse *Lsf*, cccctccagtcacggataa and gcctcgtgaatgtggagaac; human *HPRT*, tgacactggcaaaacaatgca and ggtccttttcaccagcaagct; mouse *Hprt*, tcctcctcagaccgctttt and cctggttcatcatcgctaatc; human *p21^CIP1^*, ggcagaccagcatgacagatt and gcggattagggcttcctctt; mouse *p21^CIP1^*, gaacatctcagggccgaaaa and ctcccgtgggcacttcag.

### Immunoblot analysis

Proteins were extracted from approximately 10^7^ cells with Cell-LyEx2 according to the instructions of the manufacturer (TOYO B-net). Protein concentrations were determined using a BCA Protein Assay Kit (Pierce), and equal quantities were separated under reducing conditions on precast 10% NuPAGE Novex Bis–Tris Gels (Invitrogen) with NuPAGE^®^ MOPS SDS running buffer containing 0.5% NuPAGE^®^ antioxidant. Separated proteins were then transferred to FluoroTrans^®^ PVDF transfer membranes (Pall) with the XCell II semi-wet blotting module (Invitrogen) according to the manufacturer's instructions. The membranes were blocked with 5% (w/v) nonfat dry milk in 0.05% (v/v) Tween 20-Tris-buffered saline (TBS-T) at room temperature (RT) for 30 min and then probed with the following primary antibodies diluted with Can Get Signal 1 (Toyobo): anti-LSF (ab42973, Abcam; 1:2000), anti-α-tubulin (T9026, Sigma-Aldrich; 1:10000), anti-cyclin B1 (#4135, Cell Signaling; 1:1000), anti-cyclin E (#4129, Cell Signaling; 1:1000), anti-p16 (#4826, Cell Signaling; 1:1000), anti-p21 (ab7960, Abcam; 1:1000), anti-AKT (#9272, Cell Signaling; 1:1000), anti-phosphorylated-AKT (#9271, Cell Signaling; 1:1000), anti-FAK (#3285, Cell Signaling; 1:1000) and anti-phosphorylated-AKT (#3283, Cell Signaling; 1:1000). The membranes were washed three times with TBS-T and then incubated with peroxidase-conjugated secondary anti-rabbit or anti-mouse IgG (#401315 or #401215, Calbiochem; 1:10000) at RT for 1 hour. After washing, protein bands were visualized using the Luminata Crescendo Western HRP substrate (Millipore) and captured with an LAS3000 Imager (GE Healthcare).

### Immunostaining and tissue microarray

All specimens were fixed in 4% paraformaldehyde in PBS (pH 6.8) and embedded in paraffin wax. Sections were cut at 5 μm and placed on Amino Silane-coated glass slides (Matsunami). After drying at 60°C, the slides were deparaffinized using standard protocols. Melanin was bleached following a routine method [22]. For antigen retrieval, slides were boiled in citrate buffer at pH 6.0 for 20 min and allowed to cool down to RT. Endogenous peroxidase was quenched with 3% H_2_O_2_ in methanol at RT for 20 min. After washing twice in PBS, slides were blocked with the host animal serum of the secondary antibody, incubated with the primary anti-LSF (ab42973, Abcam; 1:500), anti-Ki67 (ab66155, Abcam; 1:200), anti-DCT (sc-10451, Santa Cruz; 1:400) and anti-CD34 (ab8135, Abcam; 1:200) antibodies at 4°C for 18 h, and washed in PBS. LSF and Ki67 were detected by using the Vectastain Elite ABC Kit and diaminobenzidine (Vector Laboratories). DCT and CD34 were detected by using Alexa-488 or -568-conjugated secondary antibodies.

The human melanoma tissue array (ME1003), which contains samples from 56 cases of melanoma, 20 cases of metastatic melanoma in lymph nodes, and 24 cases of benign nevus was purchased from US Biomax and used to assess LSF expression as described above. Images were captured using an Olympus BX40 microscope, and the signal intensity was determined using WinROOF (Mitani) software. For each sample, the average intensity of the brown signal in each tumor area was calculated and LSF expression level in each tumor was classified into three groups, negative/weak, moderate or strong.

### Construction of vectors, stable cell lines and cell proliferation assay

The full-length open reading frame of *LSF* was amplified from cDNA of the normal human fibroblast cell line MRC-5 by using PrimeSTAR MAX DNA polymerase (Takara) and was cloned into pDsRed-monomer-N1 (Clontech) or pCMV-n-FLAG derived from pRK5 (BD Pharmingen) with the in-Fusion HD Cloning Kit (Takara). A 1521-bp fragment from −1520 to the transcription start site of human *p21^CIP1^* was generated by PCR using genomic DNA from MRC-5 cells and then inserted into pGL4.17 (Promega) in the same way.

SK-Mel28 and B16F10 cell clones that stably express DsRed-LSF or FLAG-LSF were generated by co-transfecting the corresponding expression constructs and a puromycin selection vector using Lipofectamine^®^ LTX (Invitrogen) and by selection with 10 μg/ml puromycin. Empty pDsRed-monomer-N1 and pCMV-n-FLAG vectors were used to establish control clones. Equal numbers (2 × 10^5^) of established cell lines were plated into 10 cm culture dishes, and the cell number was determined by hemocytometer counting at 0, 1, 2, 3, 4, 5 and 6 days.

### Transient transfection, cell proliferation assay and flow cytometry

SK-Mel28 cells plated at 1.0 × 10^5^ cells/well in six-well plates were allowed to attach to the bottom of the plate overnight and were then transiently transfected with 1-3 μg LSF-DsRed, LSF-FLAG or control vectors using Lipofectamine^®^ LTX (Invitrogen). Two days after transfection, cell growth was determined by using the crystal violet (CV) assay as described previously [23]. For knockdown of LSF, 50 pmol of LSF siRNA (Invitrogen) was transfected using Lipofectamine^®^ 2000 (Invitrogen).

For flow cytometric analysis, transiently transfected cells were harvested by trypsinization two days later, washed three times with PBS, and fixed with 70% cold ethanol before staining with 5 μg/ml propidium iodide (Sigma-Aldrich) solution in the presence of 50 μg/ml RNase A at 37°C for 30 min. At least 50,000 events were analyzed for each sample using a FACSCalibur (BD Biosciences) and the cell cycle profiles were determined using FlowJo software (Tree Star, Inc.). All experiments were performed in triplicate.

### *In vivo* tumorigenicity

A total of 10^7^ B16F10 melanoma cells stably expressing the FLAG-LSF fusion protein (sB16-FLAG-LSF cells) and control sB16-FLAG cells were subcutaneously injected into 4-week-old nude mice with a Balb/c background. Tumor growth was monitored using Vernier calipers for 5-11 weeks before sacrifice for histochemical examination.

### Electrophoretic mobility shift assay (EMSA)

Oligonucleotides and their complements (Supplemental Table 1) were synthesized (Operon) with or without biotin end-labeling. The *LSF* binding sequence from the SV40 promoter was used as a positive control [24]. After mixing, each oligonucleotide pair was boiled at 100°C and cooled down to RT for annealing. Recombinant LSF protein was prepared using the TnT SP6 Coupled Reticulocyte Lysate System (Promega). EMSA was performed using the LightShift Chemiluminescent EMSA kit (Thermo Scientific) according to the manufacturer's instructions. DNA-protein complexes were separated by electrophoresis on 7% PAGE in 0.5 x TBE buffer, transferred to Hybond-n+ (GE Healthcare), and visualized using the Chemiluminescent Nucleic Acid Detection Module (Thermo Scientific).

### Luciferase assay

SK-Mel28 cells stably expressing DsRed-LSF fusion protein and a control clone were grown to 70% confluence in 12-well plates and then transiently transfected with 900 ng of firefly luciferase reporter plasmid (human *p21^CIP1^* promoter cloned into pGL4.12) and 100 ng of Renilla luciferase control plasmid (EF-1α promoter cloned into the pRL-null vector) using Lipofectamine LTX (Invitrogen). For the siRNA experiment, 50 pmol of *LSF* siRNA (Invitrogen) was also co-transfected using Lipofectamine^®^2000 (Invitrogen). The cells were lysed 48 hrs after transfection and luciferase activity was measured by using an LB9507 luminometer (Berthold) and the Dual-luciferase Assay Kit (Promega). All experiments were performed in triplicate.

### Chromatin immunoprecipitation

Chromatin immunoprecipitation (ChIP) assays using anti-DsRed antibody (Clontech) and quantification of precipitated DNA were performed as described previously [21]. Primers used were: p21(−2000), gcgacagggctgggatctgatgc and ccagacacactctaagggaggac; p21(−1500), gcagtggggcttagagtgggg and gcagacccccttggcctgcctcg; p21(−1000), ggtagatgggagcggatagacac and gcctcctgcccggggctctctgc; p21(−500), gttggggtgtctaggtgctccag and caccgctgacccactctggcaggc; p21(−1), ggccccggggagggcggtc and gatatacaaccgccccgcc.

### Statistics

Student's *t*-test was used for statistical analysis except for the results shown in Figure 2. Fisher's exact test was used for statistical analysis of the data shown in Figure 2.

### Ethical approval

Research using human samples was approved by the ethical committees of Chubu University (approval no. 250007), Toho University (approval no. 25-1), Nagoya University (approval no. 2013-0070), Kumamoto University (approval no. 149), Nagoya Daini Red Cross Hospital (approval no. IRB20120828-1) and St. Marianna University School of Medicine (approval no. 1907). Research using animals and recombinant DNA was approved by the Animal Care and Use Committee of Chubu University (approval no. 2410061) and Nagoya University (approval no. 26317) and by the Recombination DNA Advisory Committee in Chubu University (approval no. 12-05), Toho University (approval no. G14-51-246) and Nagoya University (approval no. 13-35 and 13-76) respectively.

## SUPPLEMENTARY MATERIAL TABLE AND FIGURES


