# Supplementary material for: Transcription factor LSF (TFCP2) inhibits melanoma growth
Source: Oncotarget. 2015 Oct 25;7(3):2379–90. doi: 10.18632/oncotarget.6230 (PMC4823042; doi:10.18632/oncotarget.6230)
Supplement: Supplementary file 1 [file oncotarget-07-2379-s001.pdf]

Transcription factor LSF (TFCP2) inhibits melanoma growth

Supplementary Material

Table S1. primer sequences of the EMSA probes

Name	sequence	position
SV40 consensus	5′-CTAGCCATATGG**CTGG**TTATGG**CTGG**TCAGA-3′<br>3′-GGTATACC**GACC**AATACC**GACC**AGTCTCTAG-5′	-
SV40 consensus mutant	5′-CTAGCCATATG**TA**TG**T**TTATG**TA**TG**T**TCAGA-3′<br>3′-GGTATAC**AT**AC**A**AATAC**AT**AC**A**AGTCTCTAG-5′	-
P21 promoter #1	5′-GCACGCGAGGTT**CCGG**GACCGG**CTGG**CCTGC-3′<br>3′-CGCTCCAA**GGCC**CTGGCC**GACC**GGACGACCT-5′	-164 to -127
P21 promoter #1 mutant	5′-GCACGCGAGGTT**A**CG**T**GACCG**TA**TG**T**CCTGC-3′<br>3′-CGCTCCAA**T**GC**A**CTGGC**AT**AC**A**GGACGACCT-5′	-164 to -127
P21 promoter #2	5′-GCTGGAACTCGG**CCAG**GCTCAG**CTGG**CTCGG -3′<br>3′-CTTGAGCC**GGTC**CGAGTC**GACC**GAGCCGCGA -5′	-132 to -98
P21 promoter #3	5′-GCTGGCTCGGCG**CTGG**GCAGC**CAGG**AGCCT -3′<br>3′-CCGAGCCGC**GACC**CGTCG**GTCC**TCGGACCCG-5′	-111 to -78
P21 promoter #4	5′-GCCTCCTTGAGG**CGGG**CCCGGG**CGGG**GCGGT-3′<br>3′-GGAACTCC**GCCC**GGGCCC**GCCC**CGCCAACAT-5′	-29 to 6
P21 promoter #4 mutant	5′-GCCTCCTTGAG**TA**GG**T**CCCGG**TA**GG**T**GCGGT-3′<br>3′-GGAACTC**AT**CC**A**GGGCC**AT**CC**A**CGCCAACAT-5′	-29 to 6

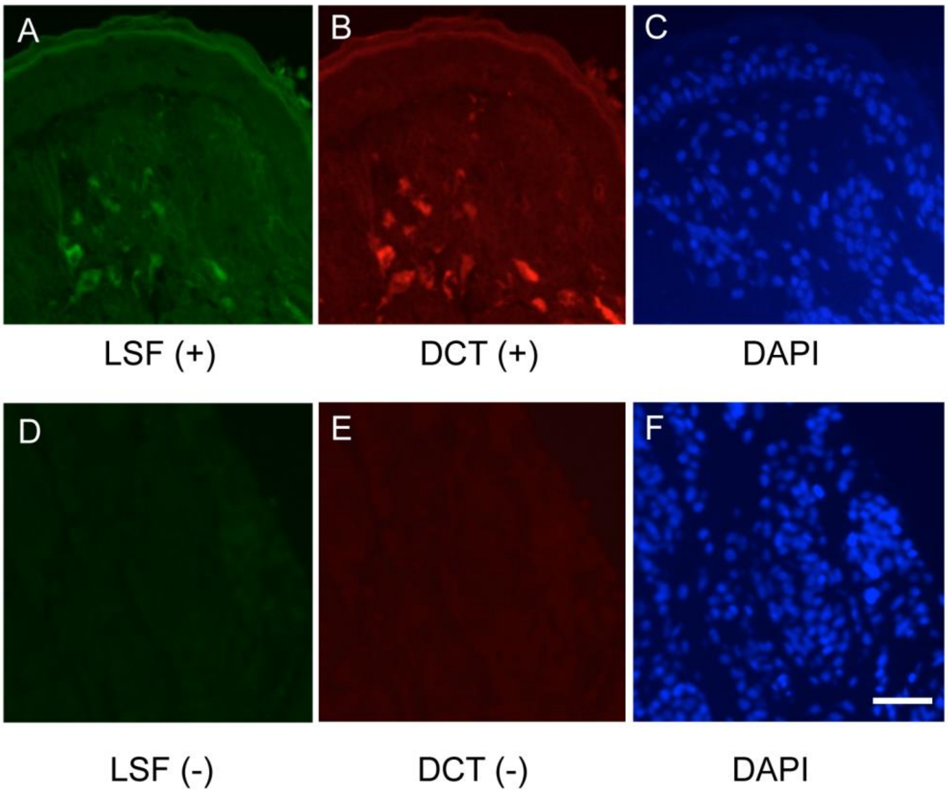

Figure S1. **Melanocyte-specific expression of LSF protein in human nevi.**

(A-F) Results for immunofluorescent detection of LSF (Green), DCT (TYRP2) (Red) and counter staining with DAPI (Blue) in human nevi are shown in the presence (A-C) or absence (D-F) of first antibodies for LSF and DCT. Co-localization of LSF and DCT shows expression of LSF protein in melanocytic cells but not in stromal cells (A-C). Expression levels of LSF and DCT were undetectably low in negative control cells (D-F). Scale bar, 100 μm.

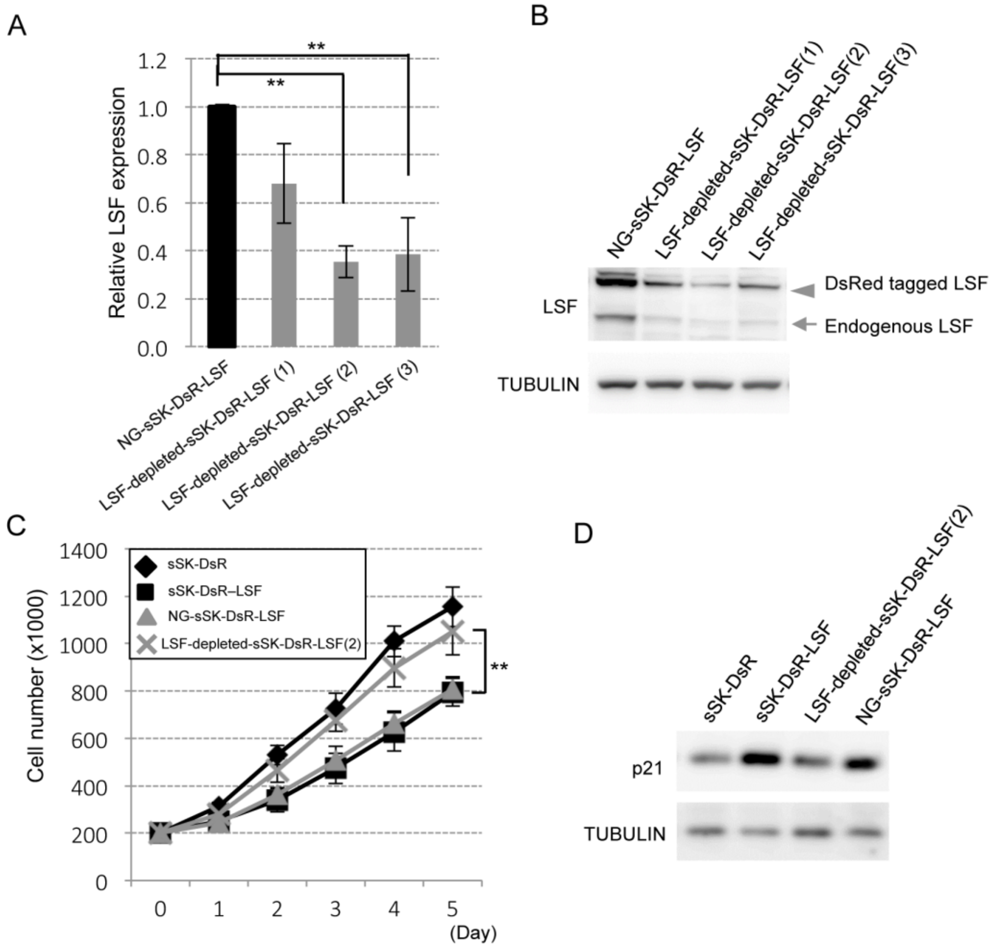

Figure S2. **Depletion of LSF expression in LSF-overexpressed SK-Mel28 melanoma cells.** (A, B) For LSF-depleted-sSK-DsR-LSF (1), (2) and (3) cells transfected with 3 kinds of siRNAs for *LSF* and NG-sSK-DsR-LSF cells transfected with siRNA for a negative control to sSK-DsR-LSF cells (clone 5), relative expression levels of *LSF* transcript (A) and protein (B) on the third day after transfection are presented. Levels of anchorage-dependent growth (mean ±SD) (C) and $p21^{CIP1}$ protein expression on the third day after transfection (D) in sSK-DsR cells (clone 9), sSK-DsR-LSF cells (clone 5), NG-sSK-DsR-LSF cells and LSF-depleted-sSK-DsR-LSF (2) cells are presented. Significantly different (**, p<0.01) from the levels of control NG-sSK-DsR-LSF cells by Student's t-test.

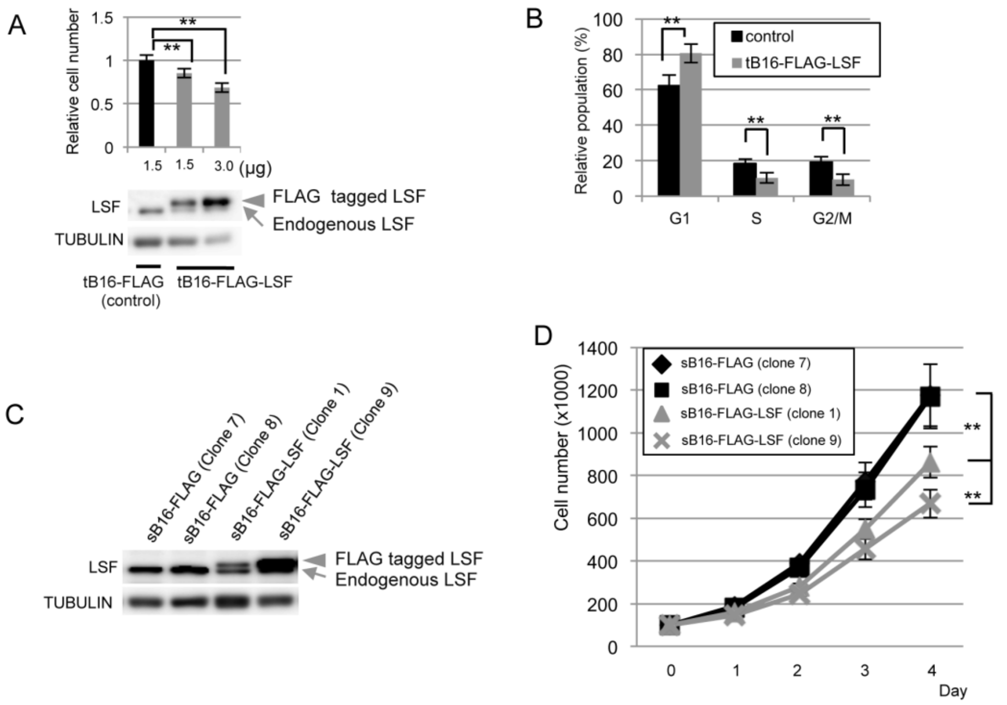

Figure S3. **Inhibition of anchorage-dependent growth by overexpression of LSF in B16F10 murine melanoma cells *in vitro*.** (A, B) After confirming LSF protein expression (bottom in A) in tB16-FLAG-LSF cells transiently transfected with the indicated amounts of the FLAG-LSF vector and control tB16-FLAG cells transiently transfected with the indicated amounts of an empty vector (top in A) on the third day after transfection, levels (means ± SD) of anchorage-dependent growth (A) and percentages of cells in G1, S, and G2/M phases (B) in tB16-FLAG-LSF cells and control tB16-FLAG cells are presented. (C, D) Expression levels of LSF protein (C) and levels (means ± SD) of anchorage-dependent growth (D) in sB16-FLAG-LSF cells stably transfected with the FLAG-LSF vector (clones 1 and 9) and control sB16-FLAG cells stably transfected with an empty vector (clones 7 and 8) are presented. Significantly different (**, $p<0.01$) from the levels of control tB16-FLAG (A, B) or sB16-FLAG (C, D) cells by Student's t-test.

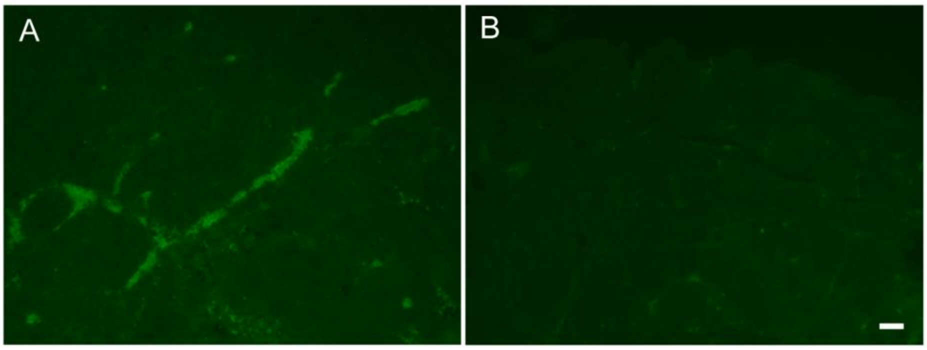

Figure S4. **Level of angiogenesis in tumors derived form LSF-overexpressed B16F10 melanoma cells.** (A, B) Representative results for immunofluorescent detection of CD34, a marker for angiogenesis, in tumors derived form control sB16-FLAG cells (A) and LSF-overexpressed sB16-FLAG-LSF cells (B). Scale bar, 100 μm.

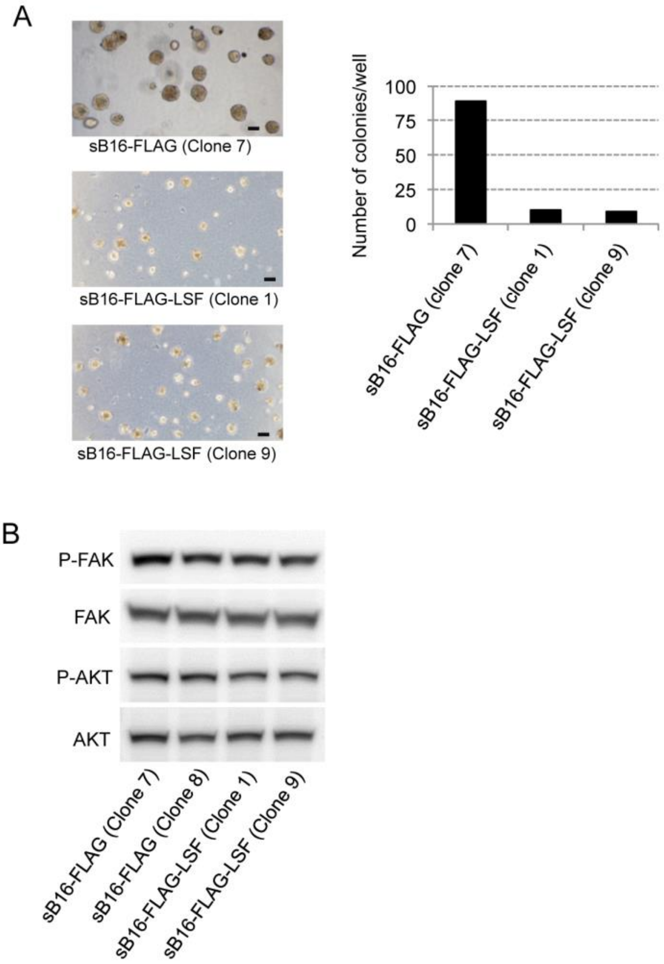

Figure S5. **Inhibition of anchorage-independent growth by overexpression of LSF in B16F10 murine melanoma cells *in vitro*.** (A) Photographs and levels (means ± SD) of anchorage-dependent growth in sB16-FLAG-LSF cells stably transfected with the FLAG-LSF vector (clones 1 and 9) and control sB16-FLAG cells stably transfected with an empty vector (clone 7) are presented. Colony formation assay was performed in 2 x 10^4 cells of each clone, which were plated per well in a medium containing 15% methylcellulose (Sigma, St Louis, MO, USA; M-0387). The number of colonies larger than 100 μm in diameter was counted after one week. Scale bar, 100 μm. Significantly different (**, $p<0.01$) from the levels of control sB16-FLAG cells by Student's t-test. (B) Phosphorylation and expression levels of FAK and AKT in sB16-FLAG-LSF cells (clones 1 and 9) and control sB16-FLAG cells (clones 7 and 8).

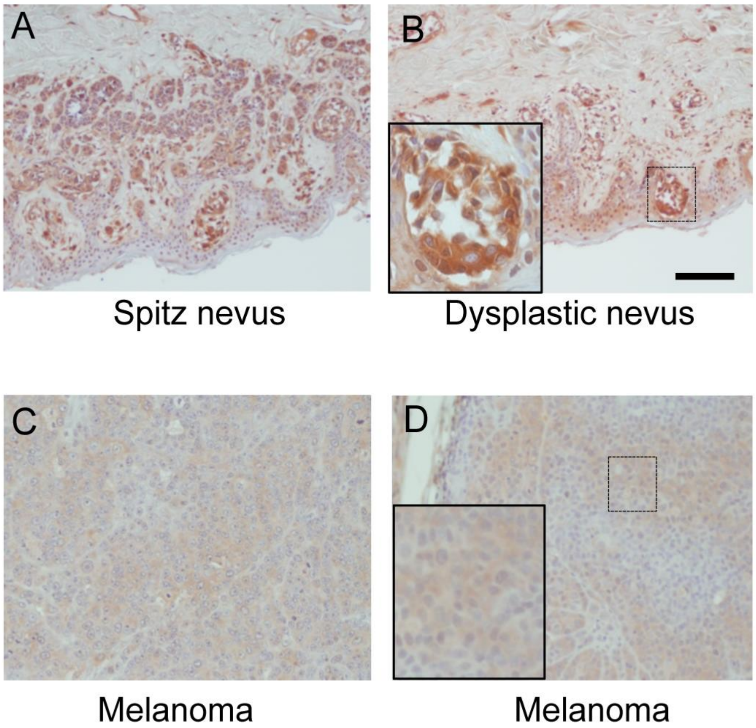

Figure S6. **Expression of LSF protein in Spitz nevus and dysplastic nevus in humans.** Results for immunohistochemical detection of LSF in human Spitz nevus (A), dysplastic nevus (B) and human melanomas (C, D) are shown. The square frame marks the area magnified in the inset. Scale bar, 100 μm.

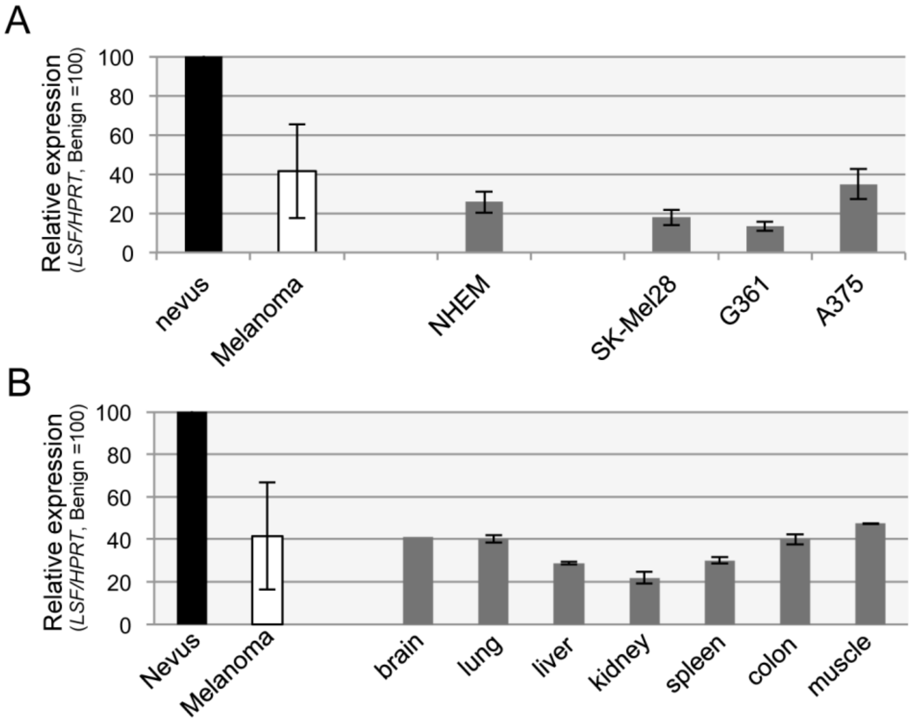

Figure S7. ***LSF* expression levels in melanoma cell lines and tissues in humans.**

Levels (mean ± SD) of *LSF* transcript expression in nevus cell nevi (n=4) (A, B), melanomas (n=10) (A, B), normal epidermal melanocytes (NHEM) (A), melanoma cell lines (SK-Mel28, G361 and A375) (A) and normal tissues in humans (B) were evaluated by RT-qPCR. Representative data of three independent experiments are presented.

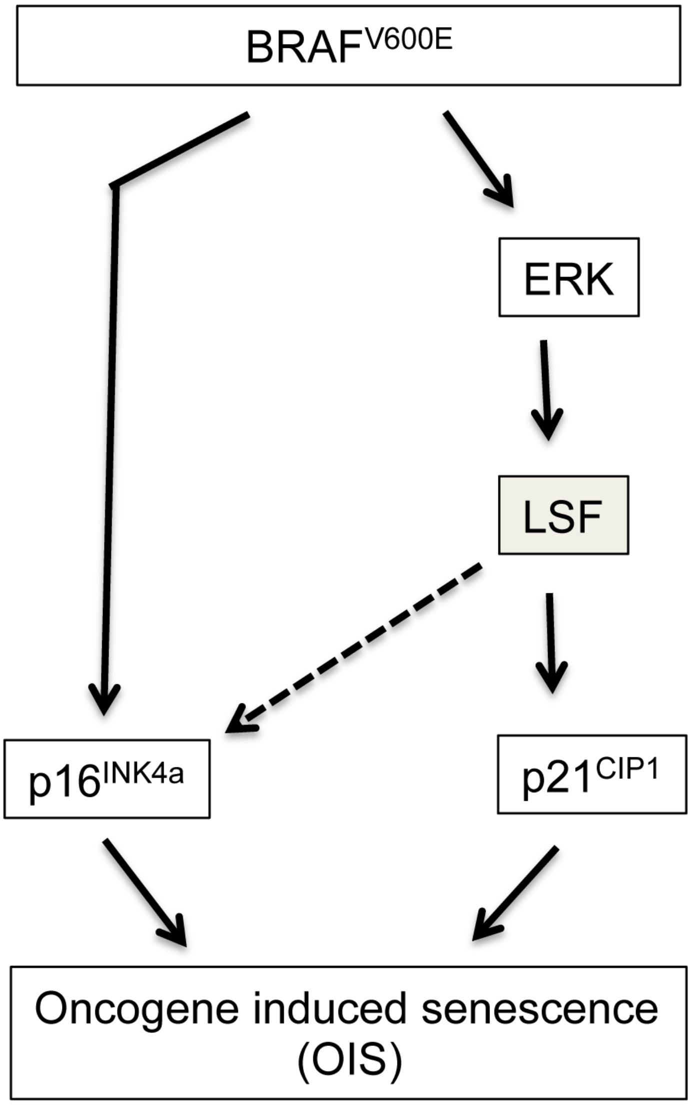

Figure S8. **Oncogene-induced senescence (OIS) in melanocytic cells via BRAF^{V600E}.**
Our proposal for the roles of LSF in melanocytic cells in the potential pathway of BRAF^{V600E}-mediated OIS is shown as a schema.
